# Soil salinity, household wealth and food insecurity in tropical deltas: evidence from south-west coast of Bangladesh

**DOI:** 10.1007/s11625-015-0337-1

**Published:** 2015-09-21

**Authors:** Sylvia Szabo, Md. Sarwar Hossain, W. Neil Adger, Zoe Matthews, Sayem Ahmed, Attila N. Lázár, Sate Ahmad

**Affiliations:** 1 Division of Social Statistics and Demography, Faculty of Social and Human Sciences, University of Southampton, Highfield Campus, Southampton, SO17 1BJ UK; 2 Department of Geography and Environment, University of Southampton, Southampton, UK; 3 Geography, College of Life and Environmental Sciences, University of Exeter, Exeter, UK; 4 International Centre for Diarrhoeal Disease Research, Bangladesh (ICDDR,B), Dhaka, Bangladesh; 5 Department of Engineering and the Environment, University of Southampton, Southampton, UK

**Keywords:** Food insecurity, Soil salinisation, Climate change, Wealth inequalities, Ganges–Brahmaputra delta, Sustainable deltas

## Abstract

As a creeping process, salinisation represents a significant long-term environmental risk in coastal and deltaic environments. Excess soil salinity may exacerbate existing risks of food insecurity in densely populated tropical deltas, which is likely to have a negative effect on human and ecological sustainability of these regions and beyond. This study focuses on the coastal regions of the Ganges–Brahmaputra delta in Bangladesh, and uses data from the 2010 Household Income and Expenditure Survey and the Soil Resource Development Institute to investigate the effect of soil salinity and wealth on household food security. The outcome variables are two widely used measures of food security: calorie availability and household expenditure on food items. The main explanatory variables tested include indicators of soil salinity and household-level socio-economic characteristics. The results of logistic regression show that in unadjusted models, soil salinisation has a significant negative effect on household food security. However, this impact becomes statistically insignificant when households’ wealth is taken into account. The results further suggest that education and remittance flows, but not gender or working status of the household head, are significant predictors of food insecurity in the study area. The findings indicate the need to focus scholarly and policy attention on reducing wealth inequalities in tropical deltas in the context of the global sustainable deltas initiative and the proposed Sustainable Development Goals.

## Introduction

Recent studies reveal that even though the hunger target of the Millennium Development Goal 1 is likely to be within reach (UN 2013), around 12 % of the global population remain deprived of food and one in eight people is suffering from chronic hunger (FAO et al. 2013). Moreover, because of the growing global population and rising consumption, it is estimated that in 2050 the demand for food could increase by more than 70 % (Royal Society 2009; World Bank 2008). Challenges of meeting this rising demand are likely to be exacerbated by long-term environmental changes in agricultural regions, interacting with demographic changes, political instability and natural disasters (Poppy et al. 2014; Smith et al. 2000). Densely populated delta regions, in the Global South in particular, will be at risk of failing to meet their global and national developmental goals, despite the declining trends of food insecurity over the past 20 years in the developing world (FAO et al. 2013; Smith et al. 2000).

Delta regions occupy 1 % of the earth’s land area and are home to more than 500 million people (Foufoula-Georgiou et al. 2011; Woodroffe et al. 2006). Because deltas constitute “rice bowls” of the world, deterioration of the tropical megadeltas poses serious threats to food security for more than half of the world’s population that relies on rice as a staple food (Hoanh et al. 2010; Pont et al. 2002). Low elevation also makes human settlement in deltas exposed to coastal flooding and storm surges (Syvitski 2008). Deltas are subject to adverse environmental changes principally through human modifications of land use over the past century, notably through rapid deforestation, urbanisation and agricultural development. Moreover, human interventions at a local level, such as dam-induced changes of river flow regime, oil extraction and groundwater extraction, influence the rate of subsidence which in turn contributes to the sinking of deltas. These changes are likely to have negative environmental and social consequences thereby putting human populations at risk of food insecurity. Some of the deltas (e.g., Ganges–Brahmaputra and Yangtze River basin) are already facing the problems of salinisation (Alam 1996) and water quality degradation (Dearing et al. 2014) which not only affects the land use and agriculture productivity of the region, but also the health and well-being of populations and the integrity of socio-ecological systems of deltas. Furthermore, soil and water salinity are projected to increase because of upstream water diversions, sea level rise and climate change (Ericson et al. 2006; Syvitski et al. 2005; Wong et al. 2014).

A number of studies have shown that higher temperatures and sea level rise have a significant effect on soil salinity, in particular in delta regions (Bazzaz et al. 1996; Gornall et al. 2010; Haider and Hossain 2013; Nicholls 2011). Tidal penetration can increase the extent of perennially and seasonally saline soils and diminish soil organic content (Bazzaz et al. 1996). Soil salinity can in turn have a negative effect on production of agricultural crops. Globally, it is expected that incidence of increase and magnitude of extreme high sea level is very likely to continue in the late twenty-first century thus exacerbating the existing threats to human livelihoods (IPCC 2013). Understanding these dynamics affecting food security is critical also in the context of the global sustainable deltas initiative called for by the scientific community (Foufoula-Georgiou et al. 2011, 2013). This initiative aims at generating and sharing knowledge on environmentally vulnerable delta regions and raising awareness of these regions.

There are, of course, well-established associations between food security and households’ socio-economic characteristics in other geographical contexts (FAO et al. 2013; Martin et al. 2004; Sraboni et al. 2014). Yet, there is limited evidence regarding these relationships in tropical delta regions despite the crucial role which deltas play in regional and global food supplies (Foufoula-Georgiou et al. 2011; Garschagen et al. 2012).

The present study hypothesises that soil salinity as well as households’ socio-economic characteristics have a direct influence on households’ food security in rural deltaic environments. More specifically, the first hypothesis states that salinisation is positively associated with household food insecurity. The second hypothesis assumes that there is an association between household’s wealth and food security. By undertaking this analysis, the main objective of the present study is to contribute knowledge regarding the determinants of food insecurity in tropical delta regions in the context of the sustainable deltas initiative and a wider sustainable development agenda.

## Background

**Fig. 1:**
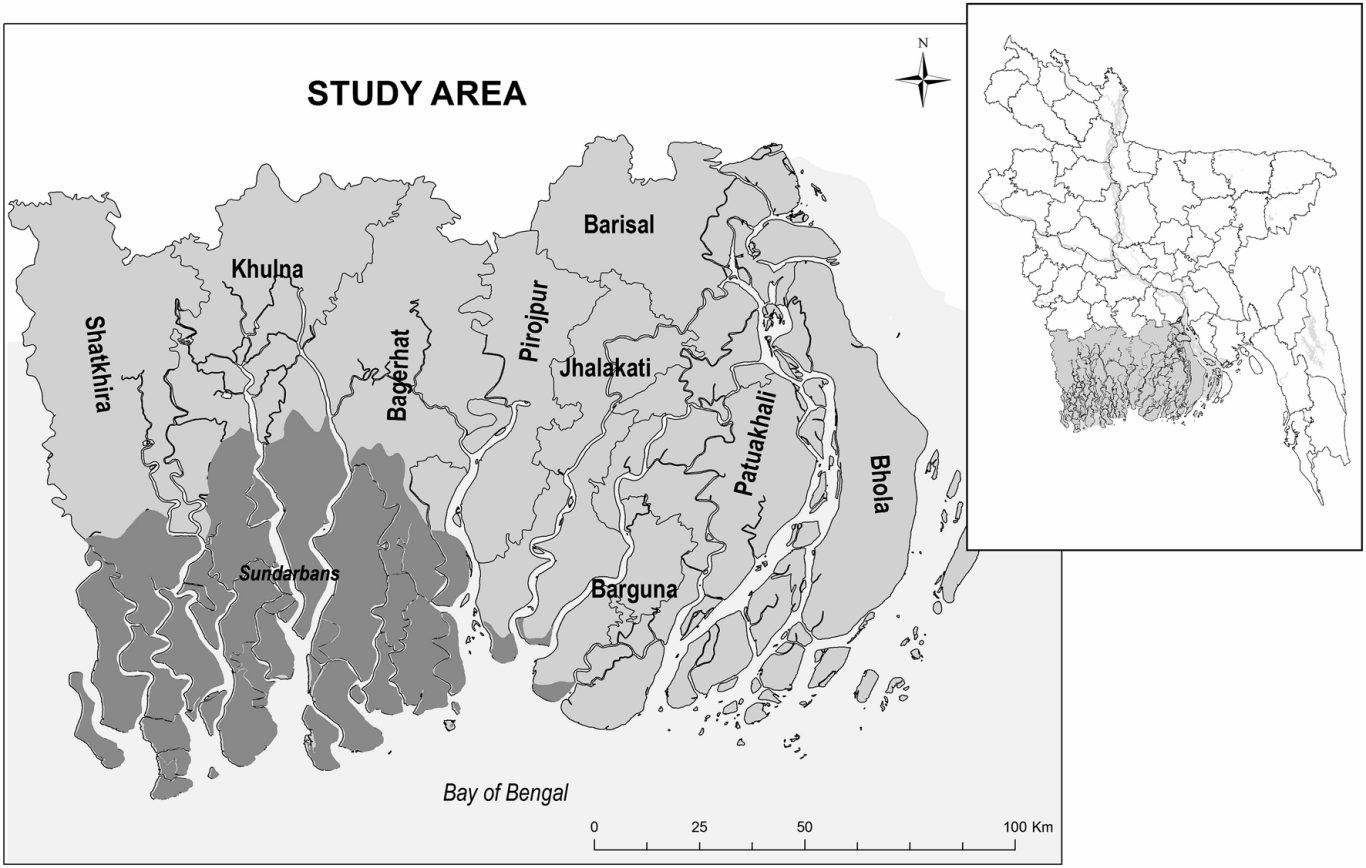
The study area in coastal Bangladesh

The study area encompasses nine districts across Barisal and Khulna divisions of Bangladesh (Fig. 1). In Khulna division, these districts are Bagerhat, Khulna and Satkhira. In the Barisal division the six districts are Barisal, Barguna, Bhola, Jhalokati, Patuakhali, Pirojpur and Barisal. As per 2011 census data, the overall population of the study area exceeds 14 million and is projected to slightly increase by 2030, if constant rates of fertility, mortality and migration are assumed (Szabo et al. 2015a, b). However, the future size and structure of the population in the region will greatly depend on future migration dynamics (Szabo et al. 2015a, b). Importantly, this densely populated delta is one of the most vulnerable regions to climate change in the world (Milliman et al. 1989). Due to sea level rise, overextraction of groundwater, upstream diversion of surface water and shrimp farming, the coastal Ganges–Brahmaputra delta has been experiencing a relatively rapid increase in groundwater salinity, river salinity and soil salinity (Dasgupta et al. 2014; Ahsan and SDRI Team 2010).

Although the coastal zone of Bangladesh is predominantly used for rice cultivation, shrimp farming is also becoming an important source of income in the study area (Chowdhury et al. 2011). Since 1970s, the international demand for shrimps accompanied by relatively high prices for shrimp products triggered increasing conversion of traditional agriculture into shrimp cultivation ponds (Rahman et al. 2013). In addition, the salt tolerance of current rice varieties is between 3 and 12 dS/m (for the dry season Boro rice varieties it is 6–12 dS/m), thus soil salinisation can also force farmers to shift from agriculture to aquaculture. Consequently, many rice fields, dominantly in the Khulna district, have been transformed into shrimp farms (“ghers”) and shrimps have become major export commodities (Ali 2006; Rahman et al. 2013). One of the main negative consequences of this changing landscape was increased water and soil salinisation gradually taking place in the region. Shrimp ponds contribute to accelerating depletion of base minerals and make adjacent soils more acid and saline, a process which is difficult to revert (Ali 2006). Between 1970 and 2010, river salinity has increased from 2 to 10 times (Hossain and Dearing 2013; Hossain et al. 2015), whereas soil salinity affected 0.223 million ha (26.7 %) during the same time period. Around 450,000 ha of coastal land were affected by salinity ingress where soil salinity exceeds 8 dS/m (SRDI 2010). Considering the above salt tolerance of rice varieties, this area is likely to be marginally productive, unless good irrigation and land management practices are in place to mitigate the effect of such soil salinity levels.

Importantly, poverty in this region is still a predominantly rural phenomenon, as is the case in other parts of Bangladesh (World Bank 2011), despite an increasing urbanisation of poverty (Planning Commission 2011). Given climate change and environmental vulnerability of the south-west coastal region, there is growing concern that households, in particular those from the poorest segments of the society, would need to develop additional coping strategies to mitigate the current and foreseen food insecurity risks (Faisal and Parveen 2004). In the absence of access to sources of financing, farmers’ livelihood strategies are likely to entail not only further conversion to shrimp farming but also increasing out-migration to urban areas, including to regions located outside of the immediate coastal area. Recent data from the 2011 Bangladeshi Population and Housing Census show that in some districts in the study area, including Khulna and Barisal, the population growth rate since the previous decennial census has been negative, indicating high out-migration rates (BBS 2012a, b).

## Conceptual framework

The conceptual framework (Fig. 1) is used to test the study’s hypotheses. While the main focus of the framework is on pathways between soil salinisation, household socio-economic characteristics and food security, it is acknowledged that these associations can also be affected by other factors, in the conceptual framework portrayed in Fig. 1. The most important mechanism is the adverse impact of salinisation on provisioning ecosystem services, such as fresh water, food and fibre. These negative impacts can be particularly strong in the absence of an adequate policy and regulatory framework resulting from weak governance structures. For example, river basin management constitutes a critical aspect of natural resource management and allows optimising the productivity of resources in the long run (Montero et al. 2006). Inadequate river basin management can lead to increased salinisation, as was for example the case in the Murray–Darling basin towards the end of the twentieth century (Squires et al. 2014). Climate change, in particular sea level rise, constitutes a threat to agricultural activities in delta regions because of salinisation of surface and ground waters leading to greater soil salinity (Nicholls 2011). Salinisation and thus high levels of soil salinity can affect households’ well-being measured by socio-economic indicators. For example, crop damage and changing patterns in crop production linked to salinity intrusion can have an adverse effect on both household livelihood strategies and outcomes.

**Fig. 2:**
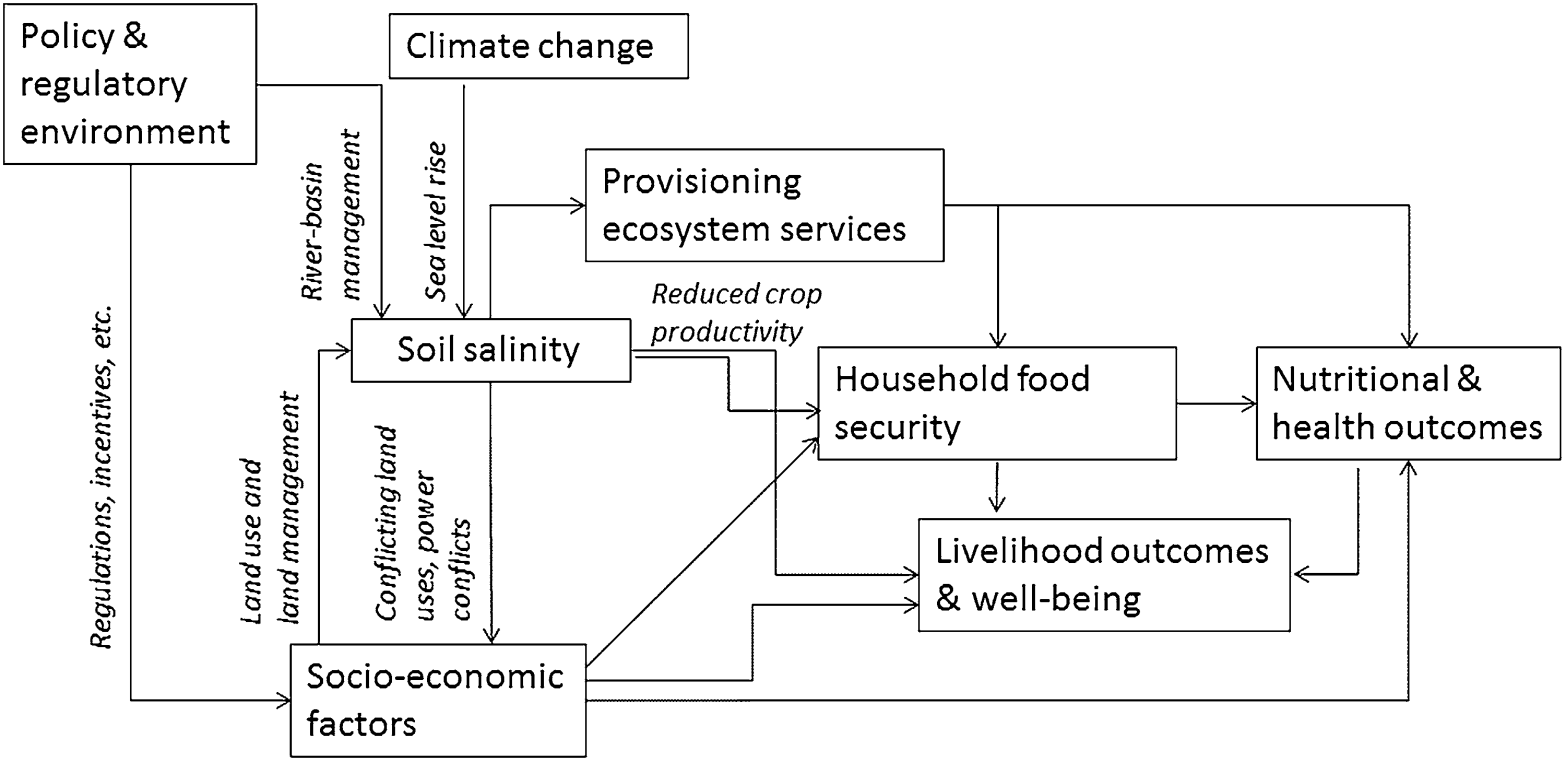
Complex mechanisms affect household food security in the coastal Ganges–Brahmaputra delta

Concurrently, socio-economic factors, such as households’ wealth can have a direct and indirect effect on household food security. Households’ wealth and education, which is an indicator of human capital (Goujon and Lutz 2004; Lutz et al. 2008), have been shown as significant determinants of food insecurity in other geographical contexts (Smith and Haddad 2000; Subramanian and Smith 2006) and are included in Fig. 2. Household food security can in turn influence nutritional and health outcomes. It has been established that malnutrition has a negative effect on correct functioning of every organ system, including muscle function and gastrointestinal function (Saunders and Smith 2010). Both household food insecurity and individual health outcomes are contributors to household livelihood outcomes and wider well-being.

In Bangladesh, analysis of secondary data from the 2011 Demographic and Health Survey (DHS) reveals that households in the highest wealth bracket (based on the quintile distribution of their assets) are considerably less likely to suffer from food insecurity compared to poorest households. Based on the indicator of frequency of skipping meals, 82 % of women in Bangladesh responded that they never had to skip a meal, while only 56 % of the poorest females were in the same situation (NIPORT et al. 2013). In addition, the proportion of those skipping meals was higher in rural areas as compared to urban areas (NIPORT et al. 2013). Given the risk of food insecurity linked to salinisation, farmers in the coastal Ganges–Brahmaputra delta have needed to adopt innovative approaches resulting in changing cropping patterns. According to a recent study investigating changing livelihood strategies in the costal delta region, 70 % of interviewed farmers from Patuakhali district stated that their shifts to different crop production were motivated by the potential for increased food security (Islam et al. 2011).

## Data and methods

### The dataset

This research makes use of the 2010 Household Income and Expenditure Survey (HIES) data as well as upazila (sub-district)-level soil salinity data developed by the Soil Resource Development Institute (Ahsan and SDRI Team 2010). The 2010 HIES followed the standard two-stage stratified random sampling procedure. The integrated multipurpose sample design included 1000 primary sampling units (PSUs) including 640 rural and 360 urban PSUs. In the Barisal division, 980 households have been selected, while in Khulna division there were 1800 sample households (BBS 2011). The analysis in the present study considers a sample of 993 households, all located in the nine rural agriculture-dominated districts of the coastal Ganges–Brahmaputra delta across Khulna and Barisal divisions.

### Key variables

#### Outcome variable

The outcome variable measures household-level food security and is based on food insecurity indicators, proposed by the International Food Policy Research Institute (IFPRI) (Smith and Subandoro 2007). This approach considers two key indicators of food security, firstly the percentage of total household expenditure on food and secondly the daily total calorie availability at the household level. A household is categorised to be food insecure if more than 75 % of its total expenditure is on food items (see also Smith and Subandoro 2007). In addition, a household is classified as food insecure if its daily calorie requirements are higher than total reported energy intake. Taking into account these two variables allow accounting for both availability and access aspects of the food security concept. A final categorisation has been developed based on the combination of the above two variables; a household is categorised as food insecure if at least one of the above conditions has not been met.

#### Explanatory variables

Key explanatory variables include households’ socio-economic characteristics, such as wealth, education, gender and engagement in agricultural activities, and upazila-level soil salinity. In addition, given the volume of remittances in Bangladesh as well as the importance of remittances for livelihoods (Adams and Page 2005; UNCTAD 2012b), a binary variable measuring whether or not a household has been receiving remittances has been incorporated into the model. Households’ wealth status has been categorised based on the asset index variable created for the purpose of this study. Although not without their limitations (Falkingham and Namazie 2002), asset indices are widely used in socio-economic analyses to approximate households’ wealth. A principal component analysis (PCA) was applied to survey responses on ownership of a set of key assets and the values of the index were based on the first principal component. The list of variables used for the creation of the asset index is provided in the “Appendix”. PCA is a commonly used technique when computing asset indices; although traditionally applied to continuous variables, Filmer and Pritchett (2001) argued that it can be a valid method for categorical and binary data such as ownership of assets. Higher scores of the index indicate more affluent households; and households can be ranked from the lowest to the highest asset score and divided into five categories to form asset quintiles. The results of Kaiser–Meyer–Olkin test of sampling adequacy (KMO = 0.67) attested that partial correlations amongst variables were high enough for the PCA to be an adequate method of data reduction in the analysis.

The household-level dataset is complemented by upazila-level soil salinity data published by Ahsan and SDRI Team (2010). This report contains field observation-based (peak) soil salinity data for 2009 for all 70 upazilas. Detailed information regarding the quality of the soil salinity data can be found in the methods section of the same report. This information enables a spatial differentiation of the salinisation problem within the coastal delta region. In the present study, two main indicators of salinisation are considered. Firstly, the extent of salinity affected areas was calculated as the percentage of saline area (2 dS/m or more) in each upazila. Secondly, a weighted average salinity score (i.e. concentration) was calculated from the soil salinity data (measured as dS/m).

### Methods

To test the hypotheses, the study uses econometric methods, including descriptive statistics and regression modelling. To compare mean salinity scores and salinity area amongst food secure and food insecure households, one-way ANOVA tests were used. Complementarily, assessing the impact of households’ socio-economic status on food security outcomes was conducted by means of χ^2^ statistics.

Because the outcome variable is binary, a series of logistic regression models were applied. The results of both unadjusted models and models which control for selected confounding factors are reported and discussed. First, the relationship between salinity affected area and households’ food security is examined. Then, selected socio-economic characteristics (not including wealth quintiles) are added. The third model controls additionally for households’ wealth status. The fourth and fifth models represent the unadjusted and adjusted relationships, respectively, between weighted salinity score and absence or presence of food insecurity in a household.

The following equation was estimated to examine the unadjusted relationship between household food insecurity and salinity intrusion:

$${\text{logit}}(Y_{i} ) = \beta_{0} + \beta_{1} X_{i} + \varepsilon_{i} \quad \quad {\text{where}}, \, i = 1, 2 ,\ldots ,{\text{n,}}$$

where *Y*_*i*_ denotes household food insecurity status with a values 0 or 1 (0 = food secure, 1 = food insecure), $\beta_{0}$ is a constant, *X*_*i*_ indicates salinity score, $\beta_{1}$ is the coefficient that shows the magnitude and direction of relationship with *Y*_*i*_ and $\varepsilon_{i}$ means error term.

The adjusted models with control variables were specified as follows:

$${\text{logit}}(Y_{i} ) = \beta_{0} + \beta_{1} X_{1i} + \beta_{2} X_{2i} + \beta_{3} X_{3i} + \beta_{4} X_{4i} + \cdots + \varepsilon_{i} ;\quad i = 1, 2 ,\ldots ,{\text{n,}}$$

where *Y*_*i*_ denotes food insecurity status with values 0 or 1 (0 = food secure, 1 = food insecure), $\beta_{0}$ is a constant, *X*_*1i*_ indicates soil salinity, $\beta_{1}$ is the coefficient that shows the magnitude and direction of relationship with *Y*_*i*_. *X*_2*i*_, *X*_3*i*_, *X*_4*i*_,… denote the control variables, for example, socio-economic characteristics, wealth quintiles and the characteristics of household’s head. $\beta_{2} , \beta_{3} ,\beta_{4} \ldots$ denote adjacent coefficients to the corresponding variables and $\varepsilon_{i}$ means error term.

The results of logistic regression are interpreted using odds ratios (OR) and associated confidence intervals (CI). An OR measures the odds of an outcome accounting for the effect of a selected explanatory variable compared with the odds of the outcome without exposure to such effect (Szumilas 2010). Confidence intervals indicate the range of plausible values for estimated ORs (Katz 2003). Standard post-estimation tests are applied to evaluate model fit and facilitate model selection. These include the likelihood ratio (LR) test, Bayesian information criterion (BIC) and Akaike Information Criterion (AIC). The results of these tests are reported in Table 2.

## Results

### Descriptive statistics

Table 1 provides an overview of descriptive statistics of key variables used in the analysis. As can be observed, a considerable proportion of population is food insecure, with almost 44.7 % of all households spending 75 % or more of their total expenditure on food and around 33.2 % having insufficient daily energy intake. In terms of salinity intrusion, the average percentage of saline area in each upazila is approximately 40 % and the overall weighted average of soil salinity is 3.62 dS/m. When considering socio-economic characteristics of households in the study area, it can be noticed that the majority of households is engaged in agricultural activities. More specifically, 81.7 % of households reported raising livestock, while 51.5 % were engaged in crop cultivation. The average age of household head was 47.6 and the average years of education of household head was 3.6. Importantly, 16.2 % of all households reported receiving either international or domestic remittances.

**Table 1:** Descriptive statistics of key variables in the analysis

Variable	Per cent (%)	Mean	SD
Food insecurity (% of food insecure HHs)			
Based on expenditure on food	44.71		
Based on calorie availability	33.23		
Based on the combination of expenditure on food and calorie availability (at least one is present)	65.56		
Salinisation			
Saline area (%)		0.40	0.28
Weighted salinity score (dS/m)		3.62	3.50
HH socio-economic characteristics			
Number of household members		4.5	1.7
Years of education of HH head		3.57	4.22
Age of HH head		47.59	14.52
HH head is female	12.39		
HH head worked during last 7 days	79.46		
HH engaged in crop cultivation	51.52		
HH raises livestock	81.72		
HH has been receiving remittances	16.20		
Overall n	993 (n)		

### Regression results

The results of regression modelling of household food insecurity are reported in Table 2. Sequential variable selection routine was applied to first test the impact of soil salinity. Overall, the results confirm research hypotheses although certain nuances should be noticed. More specifically, the results of the unadjusted model 1 suggest that a significant positive association (*p* < 0.05) exists between soil salinity and household food insecurity. When additional confounding variables are added (model 2), the impact of soil salinity remains significant, although only at 10 % significance level. As expected, the education level of the household head and involvement in agricultural activities are negatively associated with household food insecurity. In particular, education, which is an indicator of human capital (Goujon and Lutz 2004; Lutz and Goujon 2001), remains a strong predictor of food security across all models (in model 2: OR = 0.90, in model 3: OR = 0.93).

**Table 2:** Regression results with household food insecurity as the outcome variable

Food insecurity	Model 1	Model 2	Model 3	Model 4	Model 5
Variable	OR (CI)	OR (CI)	OR (CI)	OR (CI)	OR (CI)
Salinity affected area (%)	1.63 (1.02; 2.60)**	1.61 (0.97; 2.69)*	1.12 (0.65; 1.92)		
Salinity score				1.04 (1.00; 1.08)*	0.99 (0.95; 1.04)
HH socio-economic characteristics					
Wealth quintile					
Poor			0.68 (0.40; 1.09)		0.71 (0.44; 1.14)
Medium			0.35 (0.21; 0.58)***		0.33 (0.20; 0.56)***
Rich			0.28 (0.16; 0.50)***		0.26 (0.15; 0.47)***
Richest			0.26 (0.13; 0.51)***		0.27 (0.14; 0.55)***
Baseline: poorest			1.00		1.00
Religion					
Hinduism		0.73 (0.50; 1.06)	0.73 (0.49; 1.07)		0.77 (0.52; 1.14)
Buddhism		0.64 (0.10; 4.09)	0.78 (0.12; 4.98)		0.86 (0.13; 4.47)
Baseline: Islam		1.00	1.00		1.00
Number of HH members		1.07 (0.98; 1.17)	1.11 (1.01; 1.21)**		1.09 (1.00; 1.20)*
Characteristics of HH head					
HH head is female		0.65 (0.38; 1.13)	0.67 (0.37; 1.19)		0.70 (0.37; 1.26)
Baseline: HH head is male		1.00	1.00		1.00
Years of education of HH head		0.90 (0.87; 0.93)***	0.93 (0.90; 0.97)***		0.93 (0.90; 0.97)***
HH head worked during last seven days		0.76 (0.48; 1.20)	0.75 (0.46; 1.20)		0.71 (0.44; 1.16)
Baseline: didn’t work		1.00	1.00		
Age of HH head		1.00 (0.99; 1.01)	1.00 (0.99; 1.01)		1.00 (0.99; 1.01)
HH agricultural activities					
HH engaged in crop cultivation		0.78 (0.58; 1.06)	0.82 (0.60; 1.11)		0.83 (0.60; 1.14)
Baseline: HH not engaged in crop cultivation		1.00	1.00		1.00
HH raises livestock		0.65 (0.44; 0.97)**	0.69 (0.46; 1.04)*		0.73 (0.48; 1.10)
Baseline: HH does not raise livestock		1.00	1.00		1.00
Remittances					
HH has been receiving remittances		0.59 (0.39; 0.88)***	0.63 (0.41; 0.95)**		0.60 (0.39; 0.92)**
Baseline: HH has not been receiving remittances		1.00	1.00		1.00
Constant	1.53 (1.22; 1.92)***	3.78 (1.74; 8.18)***	5.32 (2.27; 12.49)***	1.59 (1.32; 1.94)***	5.70 (2.41; 13.48)***
Log likelihood	−627.5	−585.4	−560.3	−604.4	−537.4
LR test	4.2	84.9	119.6	3.6	118.4
P value	0.040	0.000	0.000	0.056	0.000
BIC	1268.8	1253.4	1230.4	1222.4	1184.0
AIC	1259.0	1194.9	1152.5	1212.7	1106.8
Number of observations	973	969	958	933	918

*p < 0.1; **p < 0.05; ***p < 0.01

Model 3 incorporates the effect of household wealth approximated by asset index. The impact of household wealth is strong; in particular when considering richest strata of the society (top three wealth quintiles are highly significant). Based on the results of model 3, *ceteris paribus*, in the study area, the odds of being food insecure for the richest households are approximately 0.26 times the odds for poorest households. As expected, household size is positively associated with food insecurity (p < 0.05), thus confirming traditional Malthusian claims regarding population pressure on natural resources. In addition, involvement in agricultural activities, especially raising livestock has an attenuating effect on household food insecurity.

An interesting and important result is that related to the impact of remittances. As highlighted previously, Bangladesh is the main receiver of remittances amongst the LDCs (UNCTAD 2012a), which is likely to affect positively well-being of receiving household members. Based on the results of model 3, the odds of being food insecure for households which have been receiving remittances are around 0.63 times the odds of being food insecure for households which have not been receiving any remittances. To explore further this effect, we performed a separate test using an unadjusted model with remittances as the only explanatory variable. The results of this model (unreported) suggested that when no other controlling factors are accounted for, the impact of receiving remittance is even stronger (OR = 0.45, p < 0.01). The results of LR, AIC and BIC tests suggest that model 3, which incorporates household wealth and other socio-economic characteristics, performs best and thus should be considered the most appropriate model amongst the first three.

As outlined in the “Data and methods” section, the study also tested for the effect of an alternative indicator of soil salinity based on a weighted average. The results including this variable are reported in models 4 and 5. This approach allowed validating the results reported in models 1–3. As can be seen, in an unadjusted model, soil salinity (i.e. weighted salinity score) is statistically significant (albeit only at 10 %). However, when other confounding factors are taken into account, in particular households’ assets, revenue from remittances and education, soil salinity is no longer statistically significant. As was the case in model 3, wealth, education and remittances are strongest predictors of food insecurity. Moreover, gender, approximated by the sex of household head is not statistically significant in either of the models. Finally, when considering the results of the LR tests and the values of BIC and AIC, it can be concluded that model 5 performs best and should thus be the preferred model.

## Discussion and conclusions

This study assessed the impact of soil salinity and household socio-economic characteristics on food security. It tested hypotheses that soil salinity is negatively associated with household food security and that households’ wealth has a positive effect on food security. The results of the present study are in line with the existing evidence pertaining to the negative impact of salinity on household food security (Parvin and Ahsan 2013). Importantly, the findings, however, show that the introduction of socio-economic characteristics, in particular household wealth, alters the nature of the association between salinity and household food security. The results suggest that household wealth, education and remittances are the most important predictors of household food security. These results complement the finding by Akter and Basher (2014) that rises in food prices have a disproportionate short-term effect on the poorest segments of the society in rural Bangladesh. The findings also highlight the importance of emerging research on migration and food security in developing countries (Azzarri and Zezza 2011; Zezza et al. 2011) and the need to further disentangle the pathways through which remittances affect micro- and macro-level food security.

Overall, the results show that salinisation of soil, as an example of long-term environmental degradation, is an important exacerbating risk, albeit well-established social determinants of food security remain crucial in addressing micro-level risks of food insecurity. Therefore, the results of the present study confirm existing research investigating similar questions. For example, a relatively recent study based on the analysis of 2005 HIES data showed that both education and wealth were significant predictors of household food security in Bangladesh (Faridi and Wadood 2010). With regard to the presupposed impact of household involvement in agricultural activities, similar findings were reported in a paper investigating nutritional and food security status in Dinajpur in northern Bangladesh. The authors found that crop cultivation and raising livestock were not associated with food security, although the models did not control for households’ wealth status (Hillbruner and Egan 2008). Finally, the insignificant effect of gender of household head resonate with the findings by Mallick and Rafi (2010) who showed that female-headed households were not significantly more insecure compared to male-headed households. This result could be explained, at least partially, by the presence of informal distributive mechanisms in Bangladesh (Mallick and Rafi 2010).

While the present study advances the scientific understanding of the determinants of food security in salinity-threatened areas, there are limitations. First, there are additional elements of salinity on well-being which are unaccounted for here. Soil salinity is affected by many external factors, including seasonality and natural hazards (Brammer 2014) and it affects well-being indirectly, through its impact on health, with those impacts being highly seasonal (Brainerd and Menon 2014). Second, environmental changes related to seasonality affect the availability of substitute income sources and informal food sources on food security at the household level, though these are difficult to capture. It is clear, for example, that shrimp collection, forest products and other food sources are important sources of nutrition for landless households at specific times of the year (Arnold et al. 2011). There is certainly evidence from Bangladesh that many ecosystem services from agriculture and delta ecosystems such as mangroves are directly affected by short-term stresses, including cyclones and storms, which interact with longer term processes, such as salinity intrusion (Shameem et al. 2014; Uddin et al. 2013). As highlighted previously, a final limitation is related to the fact that salinity is measured at upazila level, which implies that temporal and spatial inter-cluster variations are likely to exist with respect to the degree of soil salinisation. While we acknowledge that within upazila differences are likely to exist, the analysis carried out in this paper aimed to quantify the impact of aggregated soil salinity. Such an approach is important in terms of providing an overview of cross-level associations between soil salinity and food security, and consequently developing relevant policy measures. A wide body of social and environmental research recognised the significance of aggregated level data at both global (Rockstrom et al. 2009) and meso-scales (Dearing et al. 2014) and results of these studies yielded important policy implications.

From the policy perspective, it should be stressed that several official policy documents, including Perspective Plan of Bangladesh 2010–2021 (Planning Commission 2012) and Poverty Reduction Strategy (IMF 2003) explicitly state the goals to achieve universal food security in the country. Given the results here, it is crucial to recognise stark wealth-based inequalities in households’ food security in the rural Ganges–Brahmaputra delta region. With the likely increasing impact of climate change on livelihoods in tropical deltas, it is important to link both environmental and social development strategies, recognising the role that specific creeping processes, may have in food production and distribution. In this regard, the proposed Sustainable Development Goals (SDGs) constitute a move in the right direction because of the increased focus on the developmental impacts of environmental and climate change and the emphasis on societal inequalities (UN 2014; UNSC 2015). In addition, the SDGs recognise the need for resilient agricultural practices and building resilience of the poor, which is particularly relevant to tropical delta regions (Szabo et al. 2015a, b; UN 2014). Future research should therefore consider explicitly the cross-level interlinkages between socio-economic and environmental impacts on food security in the context of tropical deltas.

## Appendix: Variables used in the principal component analysis

See Table 3.

**Table 3:** Variables used in PCA

Variable	Coding	Mean
HH has electricity	1—no, 2—yes	32.5
HH has sanitary toilet	1—no, 2—yes	21.3
HH has access to improved water sources	1—no, 2—yes	89.6
Wall material	1—natural, 2—rudimentary, 3—finished	Natural—13.7; rudimentary—75.0, finished—11.3
Roof material	1—natural, 2—rudimentary, 3—finished	Natural—10.5; rudimentary- 85.8; finished—3.7
HH owns a computer	1—no, 2—yes	0.9
HH has access to internet	1—no, 2—yes	0.8
HH owns a radio	1—no, 2—yes	11.2
HH has television	1—no, 2—yes	19.5
HH has a fan	1—no, 2—yes	24.3
HH has a bicycle	1—no, 2—yes	19.9
HH has a motorcycle/scooter	1—no, 2—yes	1.7
HH occupancy status	1—squatter or other, 2—renter or free accommodation, 3—owner	Squatter or other—2.2; renter of free accommodation—7.6; owner—90.2
